# Distal radial fractures: a nationwide register study on corrective osteotomies after malunion

**DOI:** 10.1177/17531934231193849

**Published:** 2023-09-11

**Authors:** Leena Raudasoja, Heidi Vastamäki, Samuli Aspinen, Jorma Ryhänen, Sina Hulkkonen

**Affiliations:** 1 Department of Hand Surgery, Helsinki University Hospital, University of Helsinki, HUS, Finland; 2 Sports Trauma Research Unit, Hospital Mehilainen Neo, Turku, Finland

**Keywords:** Distal radial fracture, malunited fracture, malunion, corrective osteotomy, incidence

## Abstract

The aim of the present study was to explore the incidence of corrective osteotomies after conservatively treated distal radial fracture and the risk for late correction depending on the patient’s age. Based on data from the Finnish National Care Register of Health Care, Specialist Care, on all corrective osteotomies carried out in Finland during 2015–2019 in adults aged ≥20 years, we calculated the mean annual incidence rates per 100,000 person-years, standardized with the European Standard Population 2013. Using multivariable logistic regression, we calculated the risk of corrective osteotomies in various age groups. In total, 41,418 distal radial fractures were identified. Of those, 10,577 received surgical treatment in the acute phase. The incidence rate of primary operations for distal radial fractures was 47.9 per 100, 000 person-years. A total of 321 conservatively treated fractures needed corrective osteoteomy, with a surprisingly low mean annual incidence rate of 1.5 per 100,000 person-years. The risk for this was highest in patients in their fourth or fifth decade.

**Level of evidence:** III

## Introduction

Although distal radial fractures are the most common of all fracture types (Court-Brown et al., 2018), relatively little is known about the epidemiology of corrective osteotomies after the initial treatment for the fracture has failed. The treatment for distal radial fractures leads to malunion in approximately 23%–33% of patients after conservative treatment, and in 3%–10% after primary operative treatment (Katt et al., 2020; Mulders et al., 2018; Sharma et al., 2014). The typical malunion types are radial shortening, dorsal tilt, step-off at the articular surface of the radius or a combination of these (Larouche et al., 2016).

Symptomatic distal radial fracture malunions are treated operatively by either a corrective osteotomy of the radius or a shortening osteotomy of the ulna. Radial shortening leaving a positive ulnar variance of more than 3 mm significantly impairs treatment outcomes (Ng and McQueen, 2011). Therefore, in some cases, an ulna-shortening osteotomy is an option for corrective osteotomy after malunion of distal radial fractures (Terzis et al., 2020).

Only a few studies report on the incidence of corrective procedures after failed distal radial fracture treatment. Satariano and colleagues (2019) studied a US Medicare cohort of 5088 patients with a mean age of 78.3 years. In their study, 55 (1.1%) patients underwent secondary surgery at least 6 weeks after the initial fracture. Patients aged 65–69 years had a significantly higher operation rate (1.9%) compared with patients aged >80 years (0.5%). However, these results included all secondary procedures, such as distal radioulnar joint (DRUJ) procedures and wrist arthroscopy, rather than just corrective osteotomy. Dineen and colleagues (2019) identified 48,815 patients with distal radial fractures between 2007 and 2015. They found that the rates of corrective osteotomies after non-operative treatment compared with open reduction and internal fixation (ORIF) were significantly higher (0.5% vs. 0.3%). They also reported a significantly higher rate of corrective osteotomy for patients aged 50 years or older. In addition, women had a higher risk for corrective osteotomy.

Even with careful primary assessment and well-established treatment algorithms, some distal radial fracture malunions will occur. The knowledge of incidence rates for corrective osteotomy after failed fracture management is thus an important measure of the efficacy of the primary treatment. Moreover, from an economic point of view, treatment of distal radial fractures has a significant impact on both direct and indirect treatment costs. Whereas the mean cost of non-operative treatment is estimated to be less than US$500 (€447, £392) in the United States, the cost after corrective operations is increased four times (US$4152, €3708, £3253) (Dineen et al., 2019).

The aim of the present study was to explore the incidence rate of late corrective osteotomies after non-operative treatment of distal radial fractures in the entire population of Finland. The secondary aim was to study which age groups are at the highest risk for corrective osteotomy and may need careful judgement in the acute stage.

## Methods

### Registry data

The Care Register for Health Care, formerly called The Finnish National Hospital Discharge Register, is one of the oldest individual-level hospital discharge registers and covers the entire country. Its database contains comprehensive information on patient characteristics, diagnoses and any surgical procedures performed (Sund, 2012). All citizens of Finland have a personal ID number that can be linked to the registry data.

For this study, we used patient data from The Care Register for Health Care, Specialist Care, between 1 January 2015 and 31 December 2019. We included patients aged 20 years or older diagnosed with a distal radial fracture. They were identified by the International Classification of Diseases, 10th revision (ICD-10) diagnosis codes S52.5 (fracture of lower end of radius) and S52.6 (fracture of lower end of both ulna and radius). Patients with primary surgical treatment were identified with surgical procedures using the Nordic Medico-Statistical Committee (NOMESCO) classifications of Surgical Procedures (NCSP) for procedure codes (Table 1). Corrective osteotomies were identified with codes NCK30 (osteotomy of forearm bone) and NCK68 (shortening or lengthening osteotomy of forearm) accompanied by the previously mentioned diagnosis codes, and codes M84.0 (malunion of fracture) and T92.2 (sequelae of fracture at wrist and hand level).

**Table 1.** Types of surgical procedures for distal radial fracture according to NOMESCO classification.

NCJ62	Internal fixation of fracture of forearm using plate
NCJ64	Other internal fixation of fracture of forearm (screw, pin)
NCJ70	External fixation of fracture of elbow or forearm, body of ulna and radius
NDJ62	Internal fixation of fracture of wrist or hand using plate and screws
NDJ64	Internal fixation of fracture of wrist or hand wire, rod, cerclage or pin
NDJ70	Internal fixation of fracture of wrist or hand wire, rod, cerclage or pin

NOMESCO: Nordic Medico-Statistical Committee.

Permission to access the data was granted by Findata (data permit authority operating at the National Institute for Health and Welfare, THL/5178/14.02.00/2020). The research was a registry study with anonymized analysis and did not include identifiable individual participants. In accordance with Finnish legislation, no informed consent or ethical approval was sought (Medical Research Act 488/1999, amendments 295/2004, 794/2010).

### Statistical analysis

The mean crude incidence rates of distal radial fractures, their primary treatment strategy and corrective osteotomies were counted as cases per 100,000 person-years by dividing the number of new cases in each age group by the population of that age group in Finland between 2015 and 2019, for the whole adult population and in age groups of 10-year intervals from 20–29 up to ≥80 years of age. The age-standardized incidence rate was calculated as a weighted average of the age-specific incidence rates per 100,000 persons, where the weights are the proportions of persons in the corresponding age groups of the World Health Organization (WHO) standard population in 2013 (Pace et al., 2013). The 95% confidence intervals (CI) were estimated assuming a Poisson distribution of the cases. Finally, binomial logistic regression was run in the subpopulation with primarily conservatively treated distal radial fracture, aged 20–69 years, grouped in 10-year intervals, adjusted for sex and with corrective osteotomy as the outcome of interest. Odds ratios (ORs) with 95% CIs excluding 1 and *p < *0.05 were considered to be statistically significant.

## Results

The Finnish Care Register, Specialist Care, revealed a total of 41,418 cases of distal radial fractures in the adult population during the years 2015–2019. Of these, 10,577 (25.5%) were treated by primary operation and 30,841 (74.5%) non-operatively. The mean standardized incidence rate of distal radial fractures in the adult population was 137.9 per 100,000 person-years (95% CI: 137.9 to 137.9; crude incidence rate of 142.6), and for distal radial fractures treated by primary operation it was 47.9 (95% CI: 47.9 to 47.9; crude incidence rate of 48.9).

During the 5-year study period, there were 321 (1.04%) corrective osteotomies in the primarily conservatively treated group. The mean standardized incidence rate of corrective osteotomies was 1.5/100,000 person-years. Within the older age groups men aged ≥60 years and women aged ≥70 years, there was a decreasing frequency of corrective osteotomies. The total number of corrective osteotomies after operatively treated distal radial fractures was 60 (0.6%); these numbers (particularly in the age subgroups) were too low to allow a meaningful statistical analysis (Table 2).

**Table 2.** Crude incidence rates of distal radial fractures per 100,000 person-years, their primary treatment and corrective osteotomies in the adult population of Finland, 2015–2019, presented in age groups.

Primary treatment	Conservative				Operative		
Age group	*n*	Incidence rate^a^	Corrective procedures (*n*)	Incidence rate^a^ of corrective procedures	*n*	Incidence rate^a^	Corrective procedures (*n*)
20–29	2025	68.6	23	0.7	734	21.6	2
30–39	2033	57.7	42	1.2	995	28.2	3
40–49	2574	77.7	55	1.7	1340	40.5	5
50–59	5289	144.0	102	2.8	2520	68.6	19
60–69	7402	201.2	76	2.1	3039	82.6	15
70–79	6158	240.4	18	0.7	1550	60.5	6
80+	5060	341.6	5	0.3	399	26.9	0
All	30,841	142.6	321	1.5	10,577	48.9	60

a Per 100,000 person-years.

Table 3 shows the results of logistic regression analysis in the working-age population (aged 20–69 years).

**Table 3.** Binomial logistic regression analysis assessing the risk for corrective osteotomy after conservatively treated distal radial fracture in age groups (adjusted for all the variables).

Variable	OR (95% CI)	*p-*value
Sex		
Men	1 (reference)	
Women	1.23 (0.95 to 1.62)	NS
Age (years)		
20–29	1 (reference)	
30–39	1.71 (1.04 to 2.85)	**0.04**
40–49	1.86 (1.17 to 3.04)	**0.01**
50–59	1.52 (0.99 to 2.42)	NS
60–69	0.83 (0.53 to 1.34)	NS

Statistically significant values shown in bold font.

CI: confidence interval; NS, not significant; OR: odds ratio.

## Discussion

The incidence rate of corrective osteotomy after conservatively treated distal radial fracture was very low, with only 1.04% needing later correction, an incidence rate of 1.5/100,000 person-years (95% CI: 1.5 to 1.5). Our result was similar to that in the cohort of Satariano et al. (2019) of 1.1%, although they included more types of operations after failed distal radial fracture treatment. Dineen and colleagues (2019) had a similar number of patients with distal radial fracture in their study, but their incidence rate for corrective osteotomy was lower, only 0.5% after conservative treatment. One explanation might be their more active initial operative treatment percent (45.3% ORIF vs. 25.5% surgery in our study). However, the incidence of corrective osteotomy after conservatively treated distal radial fracture is low.

One reason for the low incidence of corrective osteotomies might be the nationwide treatment algorithm for distal radial fractures in the acute setting (Distal radius fracture: Current Care Summary, 2016). Earlier reports show that the incidence of operations for distal radial fractures doubled between 1998 and 2008 in Finland (Mattila et al., 2011). Since 2008, the number of operatively treated distal radial fractures has not grown at such a pace (Hevonkorpi et al., 2018), but a subtle increase from 15% to 18% was seen between 2015 and 2019 in the Finnish population in our previous study (Raudasoja et al., 2022). Similar trends can be shown internationally: steady increases in the number of operatively treated distal radial fractures have been reported from Sweden (Mellstrand-Navarro et al., 2014), the United States (Chung et al., 2009) and Korea (Jo et al., 2017).

Although some conservatively treated distal radial fractures result in malunion (Brogren et al., 2011; Katt et al., 2020; Ng and McQueen, 2011), the incidence of symptomatic malunion is not well reported. When a malunited distal radial fracture with radial shortening leads to symptomatic ulnar impaction causing ulnar pain, an ulnar shortening osteotomy can be considered a treatment choice, but is less common than radial osteotomy (Hassan et al., 2019; Stirling et al., 2023; Terzis et al., 2020).

Ours was a nationwide register study analysing the number and incidence of corrective procedures (corrective osteotomies with or without shortening or lengthening of the ulna or radius) after non-operative treatment of distal radial fractures. Our adjusted analysis of subgroups revealed that the incidence of corrective osteotomies was highest in the 30–39 and 40–49 year age groups. It seems that a malunited distal radial fracture might impair wrist function mainly at that age. In the elderly, the incidence rate decreased, which may in part be because of minor discomfort and less desire for operations because of a decreased need for power use; but it may also be because of the treating surgeons’ preferences. These patients may not be considered for corrective osteotomy and might not have indicated their need for secondary surgery for carpal tunnel syndrome or ulnar wrist pain, which were not analysed in this study.

The main strength of this study is that it covers the whole adult population of Finland. The comprehensive national register, the Care Register for Health Care, Specialist Care, has been proven to be reliable and able to identify common diagnoses accurately (Sund, 2012). The register is mandatory for all Finnish public and private hospitals and its representativeness and accuracy of coding has been shown to be reliable in other studies (Huttunen et al., 2014; Mattila et al., 2008). Lastly, because the healthcare in Finland is publicly funded, there is little, if any, financial influence on decisions to perform surgery.

The present study has some limitations. In Finland, the healthcare system consists of primary care and secondary (specialist) care. Most patients with distal radial fracture are registered with the Specialist Care Register, as emergency duty and fracture first aid care is predominantly centralized to hospital clinics in our country. However, patients treated completely in primary care or private institutions are not registered in the Care Register for Health Care, Specialist Care data. Thus, we could not avoid missing some data, which decreases reliability regarding the number of conservatively treated distal radial fractures. All surgical procedures in public as well as private institutions must be registered, and their registration is reliable. However, the side of the operation or possible reoperations on the same extremity are not distinctly recorded in the database. This may lead to underestimation if both hands were affected, as we did not account for a new osteotomy in the same or in the other hand during the 5-year period. In addition, this study was a retrospective analysis and no radiographic features were analysed. The cases were simply categorized into operative and non-operative. The indications for corrective osteotomy and the severity of symptoms could not be quantified in a registry-based study.

In conclusion, corrective osteotomies are seldom needed after conservatively treated distal radial fractures. We found the highest risk for late corrective osteotomy after conservative distal radial fracture treatment in the 30–39 and 40–49 year age groups, highlighting the importance of correct initial treatment in this age group.
